# Effect of chronic exposure to cadmium on serum lipid, lipoprotein and oxidative stress indices in male rats

**DOI:** 10.1515/intox-2015-0023

**Published:** 2015-09

**Authors:** Saeed Samarghandian, Mohsen Azimi-Nezhad, Mahmoud M. Shabestari, Farahzad Jabbari Azad, Tahereh Farkhondeh, Fereshteh Bafandeh

**Affiliations:** 1 Department of Basic Medical Sciences, Neyshabur University of Medical Sciences, Neyshabur, Iran; 2 Department of Medical Genetics, Faculty of Medicine, Mashhad University of Medical Sciences, Mashhad, Iran; 3 Preventive Cardiovascular Care Research Center, Imam Reza Hospital, Mashhad University of Medical Sciences, Mashhad, Iran; 4 Allergy Research Center, Faculty of Medicine, Mashhad University of Medical Sciences, Mashhad, Iran; 5 Department of Agriculture, Payam Noor University, Tehran, Iran

**Keywords:** cadmium, lipoprotein, oxidative stress, lipid

## Abstract

Cadmium (Cd) is an environmental toxic metal implicated in lipid abnormalities. The present study was designed to elucidate the possible association between chronic exposure to Cd concentration and alterations in plasma lipid, lipoprotein, and oxidative stress indices in rats. Sixteen male rats were assigned to 2 groups of 8 rats each (test and control). The Cd-exposed group obtained drinking water containing cadmium chloride (CdCl_2_) in the concentration of 2.0 mg Cd/L in drinking water for 3 months. At the end of the experimental period, blood samples were obtained to determine the changes of serum triglycerides (TG), total cholesterol (TC), highdensity lipoprotein cholesterol (HDL-C), low-density lipoprotein cholesterol (LDL-C), reduced glutathione (GSH), malondialdehyde (MDA) and also serum Cd contents. The results of the present study indicated that Cd administration significantly increased the serum levels of TG, TC, LDL-C, MDA and Cd with reduction in the HDL-C and GSH levels. In conclusion, evidence is presented that chronic exposure to low Cd concentration can adversely affect the lipid and lipoprotein profile via lipid peroxidation.

## Introduction

It is now well recognized that environmental problems have increased exponentially in recent decades, mainly due to the rapid growth of the human population and to an increased demand for several household materials (Samarghandian *et al*., 2013). While technological development has improved the quality of life, it has on the other hand created a number of health hazards (Samarghandian *et al*., 2013). The toxic chemicals discharged into air, water and soil get into the food chain from the environment (Syers & Gochfeld, 2000). Cadmium (Cd), one of the most important environmental and occupational toxic metals, is widely dispersed in the environment (SCPE, 2001). Cd occurs naturally in the environment from the gradual process of erosion and abrasion of rocks and soils, and from singular events such as forest fires and volcanic eruptions (Egan *et al*., 2005). In the environment, Cd is dangerous because humans consume both plants and animals that absorb Cd efficiently and concentrate it within their tissues (Talas *et al*., 2008). Depending on the dose, route and duration of exposure, Cd can damage various organs including the lung, liver, kidney, bones, testes and placenta (Pari & Murugavel, 2007). Cd is implicated in the pathogenesis of several diseases, including cardiovascular disease (CVD) as a metabolic disorder. CVD may ensue from metabolic disorders such as diabetes and dyslipidemia (Zhang *et al*., 2010). Lipid abnormalities have reached a high importance. Alterations in lipid metabolism increase the cardiovascular risk and thus the morbidity and mortality of diabetic patients (Ozturk *et al*., 2009). Quantitative and qualitative changes in the metabolism of lipids and lipoproteins have appeared in the literature. Recently, a number of studies reported on the contribution of oxidative stress to the modification of lipids and lipoproteins, which is correlated with oxygen free radical production, resulting in oxidative deterioration of lipids and proteins (Samarghandian *et al*., 2013; 2014; 2015). Recent studies have demonstrated that Cd stimulated free radical production, resulting in oxidative deterioration of macromolecules (Almasiova *et al*., 2012). The association between Cd exposure and occurrence of lipid abnormalities is well understood; however, few data have so far been published on the influence of chronic exposure to cadmium on the metabolism of lipids (Alvarez *et al*., 2007; Placer *et al*. 1966). The toxicity mechanism of Cd has been well demonstrated but the details of pathogenic mechanisms causing damage to lipid metabolism at long-time and low-dose exposure have not yet been fully elucidated. Thus, the present study was designed to determine the effect of chronic exposure to low dose of Cd on the modification of serum lipid and lipoprotein levels and also on oxidative stress indices in male rats.

## Materials and methods

### Chemicals

All chemicals and diagnostic kits were purchased from Sigma Chemical Co. (St. Louis, MO, USA).

### Animals

Sixteen male Sprague-Dawley (SD) rats, aged 9 weeks, weighing 160±15 g, were obtained from the Laboratory Animal Centre, Medical University of Mashhad. The rats were kept in their own cages, maintained in a room with 22±2 °C, 12 h light/dark cycle, and were fed standard rat chow and water ad libitum.

### Study design

After 14 days of acclimatization, the rats were randomly allocated to two experimental groups, (n=8 per group) as follows: group 1, control (C); group 2, exposed to Cd (Cd-exposed). The control group (C) was supplied pure drinking water. The experimental group (Cd) obtained drinking water containing cadmium chloride in the concentration of 20 μM; i.e., 2.0 mg Cd/L of drinking water or 200-fold of MAC (maximum acceptable concentration) for 3 months. The animals were housed according to regulations on the welfare of experimented animals. The study was conducted in Mashhad Medical University Experimental Animal Research Laboratory. Protocols were approved by the Ethical Committee. At the end of the study period, the animals were anesthetized by ether and blood was subsequently collected from the retro orbital sinus. Blood and sera were separated by centrifugation at 3,000 rpm for 10 min for assays of triglycerides (TG), total cholesterol (TC), high-density lipoprotein cholesterol (HDL-C), low-density lipoprotein cholesterol (LDL-C), reduced glutathione (GSH) and malondialdehyde (MDA).

### Biochemical analysis

#### Determination of lipid profile

Serum TG, TC, HDL-C and LDL-C were determined by using the diagnostic kit (Pars Azmoon Kit, IRI) on an automatic analyzer (Abbott, model Alcyon 300, USA). Lipid peroxidation was assessed in the serum.

#### Determination of lipid peroxidation

The levels of malondialdehyde (MDA) were measured in serum with the thiobarbituric acid reaction by the method of Placer *et al*. (1966). The quantification of thiobarbituric acid reactive substances was determined by comparing the absorption to the standard curve of MDA equivalents generated by acid catalyzed hydrolysis of 1,1,3,3 tetramethoxypropane. Every sample was assayed in duplicate, and the assay coefficients of variation for MDA were less than 3% (Tietze, 1969).

#### Determination of GSH

One hundred μl of freshly thawed plasma was pipetted into Eppendorf tubes containing 200 μl of a 10% solution of TCA (tricholoroacetic acid), vortexed and centrifuged at 4,000×g for 10 min at 10 °C. To 200 μl of the supernatant 700 μl of 400 mM Tris-HCl buffer, pH 8.9, was added, followed by the addition of 100 μl of 2.5mM DTNB dissolved in 40 mM Tris-HCl buffer pH 8.9. After 10 min at room temperature, the extinction of the samples was measured at 412 nm in a Perkin-Elmer 550S spectrophotometer. The blank consisted of DTNB instead of plasma; its extinction was subtracted from the test sample extinction before matching it with the standard curve described above (Lehnert *et al*., 1969).

#### Measurement of Cd concentration in serum

Cadmium determination in serum was performed as described by Lehnert and his colleagues (1969). Serum samples were analyzed using a graphite furnace atomic absorption spectrometer (Perkin-Elmer Mod. 2380). The light source came from a hollow cathode lamp. Accuracy was assured by three random determinations of seven different standard solutions, prepared with the same chemical reactive used during the metal analysis. For Cd, the wavelength was 283 nm and the detection limit was 0.2 μg/l. Each sample was analyzed in triplicate.

### Statistical analysis

All experiments were carried out at least in duplicate. Each group consisted of eight rats. One way analysis of variance (ANOVA) was performed and *Tukey post hoc* test was used for multiple comparisons. Statistical analyses were performed using the InStat 3.0 program. The results are expressed as mean ± SEM.

## Results

**Figure 1:**
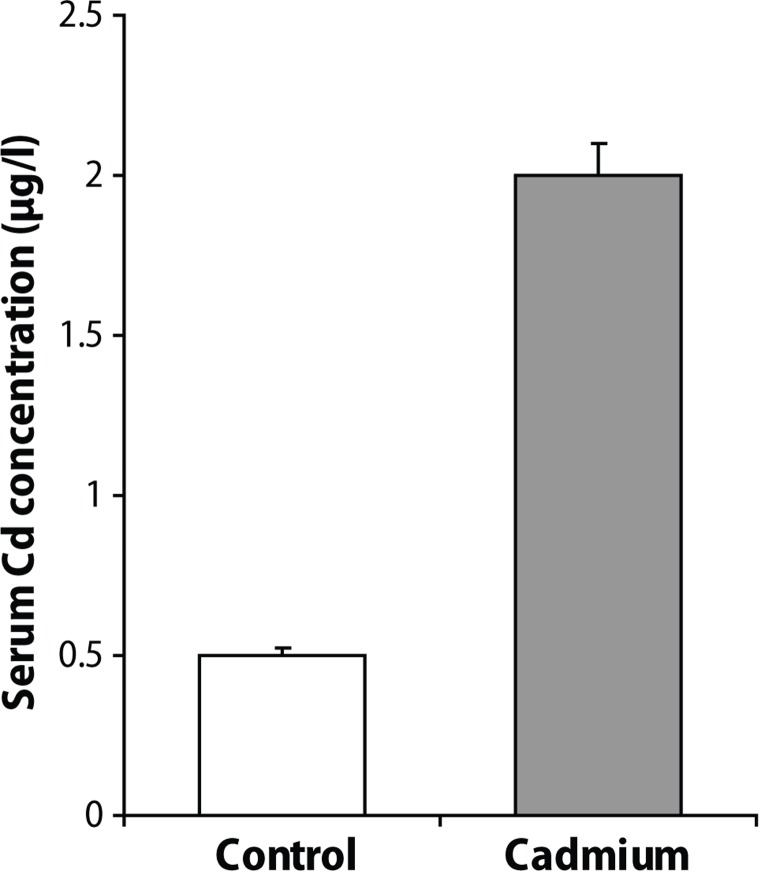
Cd concentration (μg/l) in the serum of control and Cdexposed rats. Values are the mean ± SEM. Statistical significance for the difference between the data of the control group vs CDexposed group: ***; *p* <0.001.

After the experimental period (3 months), Cd administration significantly increased the serum levels of TC, TG and LDL-C, and significantly decreased the serum HDL-C level versus the control group (*p*<0.001; *p*<0.05; *p*<0.001; *p*<0.001, respectively). Cd exposure produced significant changes in oxidative stress parameters in the serum of rats, as shown by increased MDA (*p*<0.05) and decreased the GSH content (*p*<0.05) (Table 1). The serum Cd level in the Cd-exposed group was significantly increased compared to the control group (*p*<0.001) (Figure 1).

**Table 1:** Effect of Cd on levels of lipid profile, GSH and MDA in serum of control and Cd-exposed rats.

Groups	Control	Cd-exposed group
TC (mg/dl)	34.62±3.55	53.30±5.00***
TG (mg/dl)	68.66±4.21	89.60±5.50*
LDL-C (mg/dl)	25.12±1.23	41.43±3.26***
HDL-C(mg/dl)	32.11±1.82	17±2.24***
MDA (nmol/dl)	10.11±1.81	15.3±1.10*
GSH (nmol/dl)	4.8±0.91	2.00±0.65*

Values are the mean ± SEM. Statistical significance for the difference between the data of the control group vs Cd-exposed group

* *p*<0.05

*** *p*<0.001.

## Discussion

The results of the present study indicate that oral administration of Cd significantly affected the adverse metabolic effects in rats after 3 months. Cd exposure for 3 months resulted in higher TC, TG, LDL-C, MDA and Cd levels in the serum of exposed rats compared with non-exposed animals (control). In addition, Cd administration significantly decreased HDL-C and GSH compared to control rats. The results confirmed previous findings reported by other investigators that toxic metals induce excessive amounts of reactive oxygen species (ROS) eliciting oxidative stress damage that results in many pathophysiological processes and disease development (Lehnert *et al*., 1969; Chen *et al*., 2009). Furthermore, the current results are comparable to the findings reported on Cd administration in rats, accompanied by an increase in the susceptibility to lipid peroxidation (Kara *et al*., 2007). Oxidative stress induced by oxygen derived free radicals is a disturbance in the pro-oxidant and antioxidant balance, which results in functional cell damage, undesirable biological reactions, and is involved in several pathological processes such as dyslipidemia (Olisekodiaka *et al*., 2012). A previous study indicated that chronic exposure to Cd led to increased free radical load, increased level of peroxidation products and reduced level of glutathione in rabbit models (Pathak & Khandelwal, 2007). Antioxidants are known to prevent, protect and repair free-radical-mediated damage. Reports from Pari and Murugavel proposed that diallyl tetrasulphide present in garlic attenuates the lipid peroxidation and alteration of antioxidant and membrane-bound enzymes in Cd-exposed rats (Pari & Murugavel, 2007). Olisekodiaka and co-workers confirmed previous findings that Cd can adversely affect the lipid and lipoprotein profile via lipid peroxidation (Olisekodiaka *et al*., 2012). In the current study, considerable increases in TC, TG and LDL-C fractions were observed in rats exposed to low dose of Cd as compared with the control group. Other studies have demonstrated similar increases in serum levels of TC, TG and LDL-C after high dose administration of Cd to rats (Pathak & Khandelwal, 2007). The association between Cd exposure and dyslipidemia has been confirmed by a vast amount of evidence (Badisa *et al*., 2007; Murugavel & Pari, 2007). In the present study, the plasma HDL-C concentration was significantly higher in control compared to Cd-exposed animals. HDL-C is recognized as the ‘good cholesterol’ because of its ability to reverse the transfer of cholesterol which tends to extract excess cholesterol deposited in blood vessel walls and deliver it back to the liver for catabolism (Toth & Wonger, 2003). Liver disorder resulting from exposure to Cd has been suggested to decrease the HDL-C content, to cause dyslipidemia and disturb the biological functions of HDL-C. For chronic and low-level patterns of Cd exposure that are common in humans and domestic and wild animal populations, the kidneys and liver are the primary targets of toxicity (Boisset *et al*., 1978). Although the exact mechanism underlying cadmium-induced tissue damage remains unclear, it is now largely accepted that the putative mechanism revolves around the ability of the metal to generate free radicals (Zhou *et al*., 2009) causing a change in the structure of the cellular membrane in the process of lipid peroxidation. Lipid peroxidation produced by Cd exposure was reported to induce damage to many tissues. Lipid peroxidation is one of the main manifestations of oxidative damage and has been indicated to play an important role in the toxicity of many xenobiotics (Zhou *et al*., 2009). The results of the present study confirm that chronic intoxication with Cd causes a significant increase of serum lipid peroxidation in rats. Since it causes lipid peroxidation in numerous tissues both in vivo and in vitro (Casalion *et al*., 1997; Ognjanovi *et al*., 2003; Rikans & Yamano, 2000; Tandon *et al*., 2003), Cd has been proposed to induce oxidative stress by producing hydroxyl radicals (O'brien & Salasinski, 1998), superoxide anions, nitric oxide and hydrogen peroxide. The depletion of GSH seems to be due to the generation of free radicals (Obianime & Roberts, 2009; Samarghandian *et al*., 2015). The presented results were in accordance with various previous reports suggesting that Cd can cause oxidative stress by interaction with SH-groups of the major intracellular defense glutathione (Pathak & Khandelwal, 2007). Moreover, these data confirmed increased serum lipid peroxidation in the rat by a lowered tissue GSH level during Cd intoxication (Olisekodiaka *et al*., 2012). In conclusion, this study elucidated that chronic exposure to low Cd concentration can also adversely affect the lipid and lipoprotein profile via lipid peroxidation. Further detailed studies are required for the evaluation of the exact mechanism of Cd involved in dyslipidemia.
